# Carnitine Deficiency Caused by Salcaprozic Acid Sodium Contained in Oral Semaglutide in a Patient with Multiple Acyl-CoA Dehydrogenase Deficiency

**DOI:** 10.3390/ijms26072962

**Published:** 2025-03-25

**Authors:** Yasuko Mikami-Saito, Masamitsu Maekawa, Masahiro Watanabe, Shinichiro Hosaka, Kei Takahashi, Eriko Totsune, Natsuko Arai-Ichinoi, Atsuo Kikuchi, Shigeo Kure, Hideki Katagiri, Yoichi Wada

**Affiliations:** 1 Department of Pediatrics, Tohoku University Graduate School of Medicine, 1-1 Seiryo-machi, Aoba-ku, Sendai 980-8574, Japan; 2 Department of Pharmaceutical Sciences, Tohoku University Hospital, 1-1 Seiryo-machi, Aoba-ku, Sendai 980-8574, Japan; 3 Graduate School of Pharmaceutical Sciences, Tohoku University, 1-1 Seiryo-machi, Aoba-ku, Sendai 980-8574, Japan; 4 Department of Diabetes, Metabolism and Endocrinology, Tohoku University Graduate School of Medicine, 2-1 Seiryo-machi, Aoba-ku, Sendai 980-8575, Japan

**Keywords:** carnitine deficiency, salcaprozic acid sodium, multiple acyl-CoA dehydrogenase deficiency, type 2 diabetes, oral semaglutide

## Abstract

Carnitine plays an essential role in maintaining energy homeostasis and metabolic flexibility. Various medications, such as pivalate-conjugated antibiotics, valproic acid, and anticancer agents, can induce carnitine deficiency, inhibit the utilization of fatty acid, and contribute to the development of hypoglycemia. No studies have linked oral semaglutide to carnitine deficiency. Herein, we report the case of a 34-year-old male patient with multiple acyl-CoA dehydrogenase deficiency who developed carnitine deficiency attributable to salcaprozic acid sodium (SNAC) in oral semaglutide. The patient was diagnosed with type 2 diabetes mellitus at 32 years of age and was treated with semaglutide injections. Hypoglycemic symptoms appeared after switching to oral semaglutide, and the mean levels of blood-free carnitine significantly decreased. Liquid chromatography–tandem mass spectrometry analysis revealed a peak corresponding to the SNAC–carnitine complex (*m*/*z* 423.24) in the urine exclusively during the oral administration of semaglutide. The MS/MS spectra at *m*/*z* 423.24 contained peaks consistent with those of the SNAC and carnitine product ions. Our results suggest that through complexation with carnitine, SNAC may induce carnitine deficiency. Healthcare providers should monitor for carnitine deficiency when administering SNAC-containing medications to at-risk individuals. Furthermore, this case can raise more significant concerns about the potential impact of pharmaceutical excipients like SNAC on metabolic pathways.

## 1. Introduction

Carnitine (3-hydroxy-4-N-trimethylammoniobutanoate) is an essential nutrient that plays critical roles in facilitating energy production through the fatty acids oxidation, regulating the acyl-CoA/CoA ratio within mitochondria, and excreting toxic acyl groups in the urine as carnitine esters [1,2,3,4]. Approximately 75% of body carnitine is obtained from dietary sources, with the remaining being synthesized endogenously in the liver and kidneys [5]. Skeletal and cardiac muscle contain 99% of body carnitine, with only 0.5% present in extracellular compartments, maintaining blood-free carnitine levels at 25–50 µmol/L [6,7,8]. More than 90% of urine-excreted carnitine is reabsorbed by the kidneys when blood-free carnitine levels are within the normal range [9]. By regulating carnitine excretion, the kidneys maintain blood carnitine concentration within physiological limits [10]. Body carnitine homeostasis relies on the coordination of dietary intake, endogenous synthesis, renal excretion, and reabsorption. Carnitine deficiency results from the disruption of any of these metabolic processes and is defined as an insufficient concentration of carnitine in tissues and blood, thereby impairing proper organ function. The underlying causes classify the primary or secondary types of carnitine deficiency, and among them, secondary carnitine deficiency can be induced by medications, including pivalate-conjugated antibiotics, valproic acid, and various anticancer drugs [6,11,12,13]. Pivalate-conjugated antibiotics contain pivalic acid to enhance intestinal absorption; however, pivalate binds to free carnitine in the blood, increasing urinary carnitine excretion and leading to carnitine deficiency [11]. Although the effect of pivalate on carnitine homeostasis might be negligible in healthy adults, it can contribute to carnitine deficiency in vulnerable populations, such as infants, individuals with chronic renal or hepatic insufficiency, and those with inherited metabolic disorders, leading to hypoglycemia and acute encephalopathy [14,15,16,17,18].

Multiple acyl-CoA dehydrogenase deficiency (MADD, MIM#231680), also known as glutaric acidemia type II, is a fatty acid oxidation disorder (FAOD) caused by defects in either electron transfer flavoprotein (ETF) or ETF dehydrogenase [19]. ETF is a heterodimeric protein in the mitochondrial matrix that accepts electrons from acyl-CoA dehydrogenase involved in fatty acid β-oxidation, as well as from various other dehydrogenases participating in amino acid, choline, and other metabolic pathways [20]. The electrons are subsequently transferred to ETF dehydrogenase, situated in the inner mitochondrial membrane, and then to the respiratory chain [21]. Consequently, MADD impairs fatty acid oxidation, resulting in acylcarnitine accumulation. Accumulated acylcarnitine can inhibit the organic cation/carnitine transporter 2-mediated transport and increase the urine excretion of carnitine, resulting in carnitine deficiency [6,22]. Treatment for individuals with MADD includes frequent high-carbohydrate, low-protein, and low-fat diet meals, along with riboflavin, carnitine, and coenzyme Q10 supplementation [23].

Salcaprozic acid sodium (SNAC), a synthetic N-acetylated amino-acid derivative of salicylic acid, functions as an intestinal permeation enhancer similar to pivalic acid [24,25]. SNAC, which exhibits weakly acidic properties with amphiphilicity and surface activity, was discovered through screening for substances capable of chaperoning drugs with low mucosal permeability across the intestine [26]. SNAC was incorporated into an oral formulation of vitamin B_12_, which was approved as a medical food in 2014 [27]. In 2019, the first oral glucagon-like peptide-1 receptor agonist, semaglutide formulated with SNAC, was developed and approved by the Food and Drug Administration [28]. The pH buffering effect of SNAC in the stomach prevents semaglutide degradation, enhances its solubility, and promotes its monomerization, thus increasing its permeability through the gastric mucosa [29]. Oral semaglutide demonstrates safety and tolerability identical to those of subcutaneous semaglutide, and no studies have linked it to carnitine deficiency [28,30].

In this study, we report a case of MADD presenting with carnitine deficiency attributed to oral semaglutide and identify SNAC as a potential inducer of carnitine deficiency.

## 2. Results

### 2.1. Case Description

A 34-year-old man with MADD first presented with consciousness disturbance, hypoglycemia, acidosis, and hyperammonemia when he developed acute gastroenteritis at the age of 1 year. Urine organic acid analysis revealed elevated levels of ethylmalonic acid and glutanic acid. Genetic testing identified heterozygous pathogenic variants in the *ETFA* gene (NM_000126.4): c.[764G>T];[478delG], confirming MADD [31]. The patient was treated with riboflavin, levocarnitine, and allopurinol. Despite experiencing occasional hypoglycemic episodes during infancy, the patient had an uneventful course after adolescence.

At the age of 32 years, the patient was diagnosed with type 2 diabetes mellitus and began treatment with a once-daily liraglutide injection [32]. Treatment was changed to weekly semaglutide at the age of 33 years and 2 months to improve convenience. Owing to the temporary discontinuation of manufacturing and export of semaglutide injectable formulations, the patient transitioned to daily oral semaglutide at the age of 33 years and 8 months. Four months after this transition, the patient presented with headaches and vomiting. Blood glucose and free carnitine levels were 3.3 mmol/L and 19.7 μmol/L, respectively (Table 1). The mean free carnitine level during oral semaglutide administration was significantly lower than that during semaglutide injection (29.3 vs. 63.8 μmol/L, *p* = 0.012) (Figure S1). We observed no changes in medication adherence, dietary habits, body weight, or hepatic and renal functions. After increasing the levocarnitine dose from 1500 to 3000 mg and returning to semaglutide injection, the hypoglycemic symptoms resolved, and free blood carnitine levels increased (Table 1).

### 2.2. MS Analysis Results

**Figure 1:**
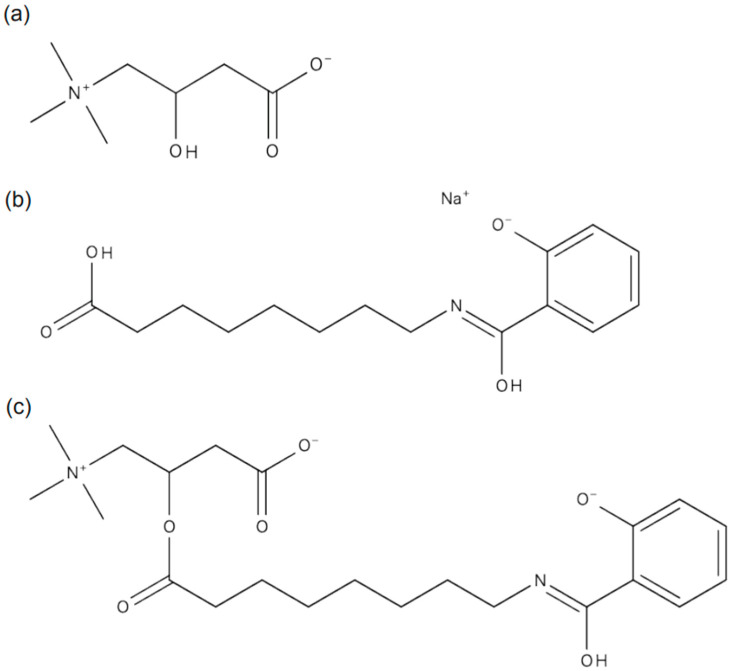
Chemical structures of (**a**) carnitine, (**b**) salcaprozic acid sodium (SNAC), and (**c**) SNAC–carnitine complex.

**Figure 2:**
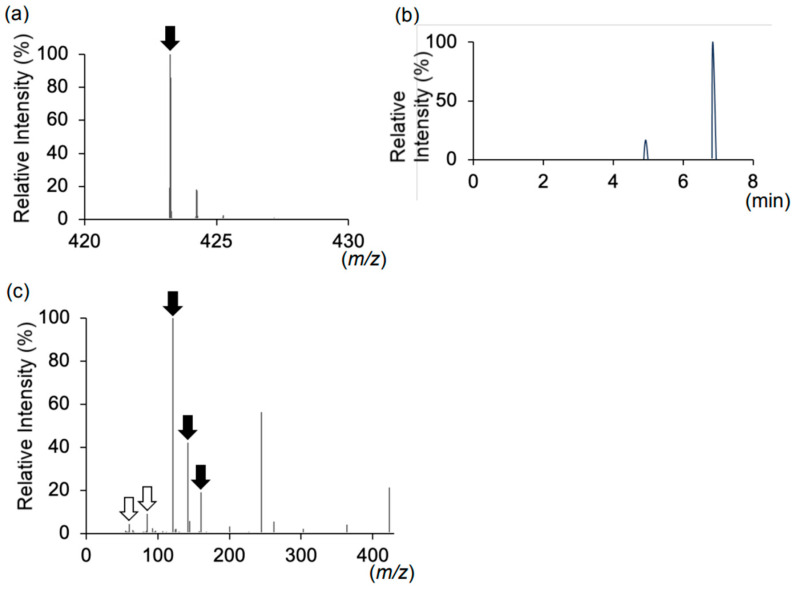
(**a**) MS spectra of *m*/*z* 420–430 in the urine during the period of oral semaglutide administration. (**b**) The chromatogram of *m*/*z* is 423.24. (**c**) MS/MS spectra of *m*/*z* 423.24, corresponding to the molecular weight of the SNAC–carnitine complex. The closed arrows indicate the product ions of SNAC, and the open arrows indicate those of carnitine.

The period during which the patient developed carnitine deficiency coincided with the oral administration of semaglutide (Table 1), leading us to hypothesize that oral semaglutide may cause carnitine deficiency. Given the molecular structure of oral semaglutide, we considered the possibility of dehydration condensation between SNAC and free carnitine in the blood (Figure 1). We performed liquid chromatography–tandem mass spectrometry (LC-MS/MS) analysis on plasma, serum, and urine samples to investigate this hypothesis. LC-MS/MS analysis revealed a peak corresponding to the molecular weight of the SNAC–carnitine complex (*m*/*z* 423.24) in urine during oral semaglutide administration (Figure 2a, Table S1). Although the *m*/*z* 423.24 peak eluted at 4.9 and 6.8 min (Figure 2b), both peaks exhibited identical MS/MS spectra (Table S2). The MS/MS spectra at *m*/*z* 423.24 contained fragment ions corresponding to carnitine and SNAC product ions (Figure 2c, Table S3). We were unable to identify the corresponding peak for the SNAC–carnitine complex in serum or plasma samples.

## 3. Discussion

We report a case of carnitine deficiency in a patient with MADD treated with oral semaglutide. The patient, who had developed type 2 diabetes, was initially treated with semaglutide injections. Upon transitioning to oral semaglutide, the patient developed hypoglycemic symptoms accompanied by markedly reduced blood-free carnitine levels. Our LC-MS analysis detected a peak corresponding to the mass-to-charge ratio of the SNAC–carnitine complex exclusively in urine during oral semaglutide administration. The SNAC–carnitine complex eluted at 4.9 and 6.8 min, with both peaks exhibiting identical MS/MS spectra, suggesting they represent structural isomers. MS/MS analysis confirmed the presence of product ions corresponding to SNAC and carnitine. These findings suggested that carnitine deficiency resulted from SNAC and carnitine complex formation. Healthcare providers should monitor for SNAC-induced carnitine deficiency, particularly in vulnerable individuals.

Our study demonstrated that SNAC likely undergoes dehydration and condensation with free carnitine in the blood, forming a SNAC–carnitine complex, which leads to secondary carnitine deficiency. This finding is supported by the temporal association between the onset of the carnitine deficiency and the initiation of oral semaglutide treatment, as well as the recovery of blood carnitine levels after discontinuing oral semaglutide. No significant changes in the diet, body weight, medication adherence, or liver and kidney functions of the patient, ruling out alternative explanations for the carnitine deficiency. Oral semaglutide improves its absorption in the gastrointestinal tract by preventing enzymatic degradation in the stomach, a process facilitated by SNAC, which increases the local pH of the stomach [29]. SNAC, containing a fatty acid moiety, is rapidly absorbed in the stomach and excreted. In this study, the SNAC–carnitine complex was detected in urine, suggesting that stomach-absorbed SNAC undergoes conversion by acyl-CoA synthetase into a CoA ester of salcaprozate before excretion. The estimated quantity of SNAC entering systemic circulation aligned with the observed reduction in plasma carnitine levels, considering that the oral bioavailability of SNAC in monkeys has been reported to be approximately 15% [26]. However, the serum or plasma did not detect the SNAC–carnitine complex peak. Previous studies have shown the rapid absorption and elimination of semaglutide-contained SNAC, characterized by a short residence time after administration [29,33]. The absence of the SNAC–carnitine complex in the blood could be attributed to its high clearance rate, suggesting that it is excreted before blood collection. To further substantiate the relationship between SNAC and carnitine deficiency and clarify the clinical significance of the SNAC–carnitine complex from a different perspective, comprehensive data on carnitine profiles should be collected from larger and more diverse populations treated with SNAC-containing medications.

Healthcare providers should exercise vigilance regarding carnitine deficiency when administering SNAC-containing drugs to at-risk patients. Secondary carnitine deficiency caused by pivalate-containing medications occurs most commonly in 1-year-old infants because of their low biosynthesis, limited muscle volume, and unstable intake; however, older individuals with underlying medical conditions can also develop this condition [15,16]. For instance, individuals with type 2 diabetes, particularly those with diabetic complications, face an increased likelihood of carnitine deficiency and may develop additional risk factors, such as hemodialysis-requiring renal failure and nonalcoholic fatty liver disease, further exacerbating the condition [34,35,36]. Similarly, organic acidemias and FAODs may contribute to carnitine deficiency by lowering the renal threshold for carnitine and increasing renal carnitine excretion. As more individuals with inherited metabolic disorders survive into adulthood, the prevalence of adult-onset complications, such as diabetes, is expected to increase [37]. This trend suggests the potential emergence of previously unrecognized drug-related side effects, as observed in this case. In addition to SNAC, medium-chain fatty acids such as sodium caprylate and sodium caprate are utilized as permeation enhancers [38]. Given their structural similarity to SNAC, agents containing these substances could potentially also induce carnitine deficiency. Therefore, blood carnitine levels should be monitored following medication administration or changes in individuals at risk for carnitine deficiency, regardless of whether symptoms are present. No previous studies have linked oral semaglutide to carnitine deficiency induced by the use of oral semaglutide [30]. In this case, the diagnosis of carnitine deficiency was facilitated by the MADD condition of the patient and routine acylcarnitine profile measurements. In contrast, in individuals with diabetes experiencing hypoglycemia, medication side effects are often suspected first, potentially reducing opportunities to check blood carnitine levels. Quantitative analysis of carnitine and the SNAC–carnitine complex in the urine and blood of individuals administered SNAC-containing medications could provide insights into several aspects, such as the duration and dosage of SNAC-containing agents required to induce carnitine deficiency, as well as the characteristics of individuals susceptible to this condition.

This study has some limitations. First, this result was based on data from only a single case experienced at our institute. Second, we did not conduct a quantitative analysis of free carnitine or the SNAC–carnitine complex in the urine. Simultaneous and longitudinal quantification of these compounds would help to support the complex formation that induces carnitine deficiency. Specifically, such an analysis could clarify the extent of carnitine loss through renal excretion, thereby allowing for the assessment of the potential clinical significance and risk of carnitine deficiency in a practical and clinically relevant manner. In addition, conducting such studies in a more diverse population receiving oral semaglutide—with stratification by renal function and assessment of carnitine deficiency risk across different renal function levels—would further strengthen the evidence for the association between SNAC and carnitine deficiency.

## 4. Materials and Methods

Plasma, serum, and urine samples were collected during oral and subcutaneous semaglutide administration. Carnitine and SNAC were purchased from FUJIFILM Wako Pure Chemical Corporation (359-44361, Osaka, Japan) and Targetmol (T8926, Boston, MA, USA), respectively.

Samples (10 μL), carnitine (10 μL, 100 μmol/L), and SNAC (10 μL, 100 μmol/L) were added to the internal standard solution (10 μL) and acetonitrile (70 μL), and then centrifuged at 15,000× *g* for 5 min. The 90 μL of the supernatant was transferred to another tube, evaporated at 40 °C for 35 min, and dissolved in 20 μL of 50% methanol.

A hybrid quadrupole time-of-flight tandem mass spectrometer (TripleTOF 5600; SCIEX, Framingham, MA, USA) was used in conjunction with an ultrahigh-performance liquid chromatograph (Nexera; Shimadzu, Kyoto, Japan). A capcell pak ADME column (50 mm × 2.1 mm i.d., 2 μm) was used, with the temperature maintained at 40 °C. Measurements were conducted in the positive ion mode. Mobile phases consisted of (A) formic acid/water (0.1:100, *v*/*v*) and (B) formic acid/acetonitrile (0.1:100, *v*/*v*). The flow rate was 0.3 mL/min, with an injection volume of 5 μL.

Mean carnitine levels during semaglutide injection and oral semaglutide administration periods were compared using the Mann–Whitney U test. The data were analyzed with GraphPad Prism 10.0.2 (Dotmatics, Boston, MA, USA), and statistical significance was defined as *p* < 0.05.

## 5. Conclusions

In conclusion, this case highlights the potential for secondary carnitine deficiency caused by SNAC-containing agents in individuals at risk. A thorough investigation, considering the medical history of the individual and the excipients of the administered drug, explained the underlying causes of atypical or seemingly incomprehensible symptoms. Further research is required to establish the association and consequences between SNAC and carnitine deficiency by collecting and analyzing comprehensive data across diverse populations. Pharmaceutical excipients should be given attention for their potential to induce unexpected metabolic effects.

## Figures and Tables

**Table 1:** Clinical data before and after carnitine deficiency: body weight, acylcarnitine profile, hypoglycemic symptoms, and medications.

Months Relative to the Episode	−8	−6	−4	−2	0	2	4	5	6	8
Body Weight (kg)	81.5	81.7	81.3	80.5	80	81.3	77.9	76.8	75	78.2
Acylcarnitines in dried blood spots (μmol/L)										
C0	74.92	68.68	63.8	48.69	19.7	34.45	14.29	69.87	83.73	66.02
C2	7.63	7.14	7.09	6.3	3.33	4.48	4.3	6.87	10.56	13.42
C4	0.75	0.69	0.91	0.62	0.35	0.45	0.38	0.94	1.05	1.11
C6	0.49	0.32	0.7	0.66	0.18	0.26	0.17	0.36	0.92	1.19
C8	0.87	0.3	1.19	1.31	0.32	0.44	0.33	0.5	1.44	2.35
C10	0.37	0.12	0.45	0.47	0.17	0.2	0.14	0.19	0.62	1.17
C12	0.07	0.04	0.08	0.07	0.05	0.05	0.04	0.06	0.11	0.14
C14:1	0.09	0.04	0.08	0.06	0.06	0.07	0.06	0.07	0.14	0.14
C16	0.46	0.33	0.5	0.42	0.38	0.48	0.36	0.47	0.55	0.61
Hypoglycemic symptoms	−	−	−	−	+	+	+	−	−	−
Treatment										
Semaglutide injection (mg/week)	0.5	0.5	−	−	−	−	−	0.25	0.25	0.25
Oral semaglutide (mg/day)	−	−	3	7	7	7	7	−	−	−
Levocarnitine (mg/day)	1500	1500	1500	1500	1500	1500	1500	3000	3000	3000

C0, free carnitine; −, absent; +, present.

## Data Availability

The data of this study are available within the article and in Supplemental Figures and Tables. The clinical data were de-identified.

## Supplementary Materials

The following supporting information can be downloaded at https://www.mdpi.com/article/10.3390/ijms26072962/s1.
