# Supplementary figures and images for: Carnitine Deficiency Caused by Salcaprozic Acid Sodium Contained in Oral Semaglutide in a Patient with Multiple Acyl-CoA Dehydrogenase Deficiency
Source: Int J Mol Sci. 2025 Mar 25;26(7):2962. doi: 10.3390/ijms26072962 (PMC11989126; doi:10.3390/ijms26072962)

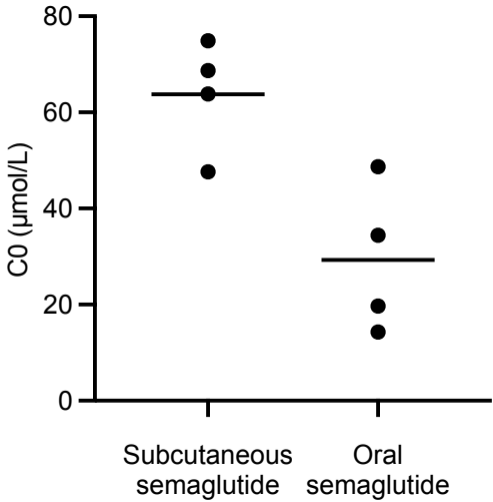

Supplement: Supplementary file 1 [file ijms-26-02962-s001.zip › ijms-3468838-supplementary.pdf]
